# The Gastroprotective Effect of Walnut Peptides: Mechanisms and Impact on Ethanol-Induced Acute Gastric Mucosal Injury in Mice

**DOI:** 10.3390/nu15234866

**Published:** 2023-11-22

**Authors:** Yutong Yuan, Xinyi Wang, Yumeng Wang, Yaqi Liu, Liang Zhao, Lei Zhao, Shengbao Cai

**Affiliations:** 1 Beijing Engineering and Technology Research Center of Food Additives, School of Food and Health, Beijing Technology and Business University, Beijing 100048, China; s20223061183@cau.edu.cn (Y.Y.); xaesi23@163.com (X.W.); liuyq2617@163.com (Y.L.); zhaolei@th.btbu.edu.cn (L.Z.); 2 Beijing Key Laboratory of Functional Food from Plant Resources, College of Food Science and Nutritional Engineering, China Agricultural University, Beijing 100083, China; goodbai0427@163.com; 3 Faculty of Food Science and Engineering, Kunming University of Science and Technology, Kunming 650500, China

**Keywords:** walnut peptides, gastric mucosal injury, LC-MS/MS, alcohol-induced, Western blot

## Abstract

The objective of this research was to explore the protective impact of walnut peptides (WP) against ethanol-induced acute gastric mucosal injury in mice and to investigate the underlying defense mechanisms. Sixty male BALB-c mice were divided into five groups, and they were orally administered distilled water, walnut peptides (200 and 400 mg/kg bw), and omeprazole (20 mg/kg bw) for 24 days. Acute gastric mucosal injury was then induced with 75% ethanol in all groups of mice except the blank control group. Walnut peptides had significant protective and restorative effects on tissue indices of ethanol-induced gastric mucosal damage, with potential gastric anti-ulcer effects. Walnut peptides significantly inhibited the excessive accumulation of alanine aminotransferase (ALT), aspartate transferase (AST), and malondialdehyde (MDA), while promoting the expression of reduced glutathione (GSH), total antioxidant capacity (T-AOC), glutathione disulfide (GSSG), and mouse epidermal growth factor (EGF). Furthermore, the Western blot analysis results revealed that walnut peptides significantly upregulated the expression of HO-1 and NQO1 proteins in the Nrf2 signaling pathway. The defensive impact of walnut peptides on the gastric mucosa may be achieved by mitigating the excessive generation of lipid peroxides and by boosting cellular antioxidant activity.

## 1. Introduction

As the economy grows, the rising popularity of alcoholic drinks coincides with numerous studies indicating that excessive alcohol intake can have detrimental effects on one’s well-being [1]. Under normal circumstances, the gastric mucosal barrier is composed of factors such as the mucus–bicarbonate–phospholipid barrier, the epithelial barrier, and the endothelial barrier, which work together to protect the integrity and functionality of the gastric mucosa [2]. When ingested in high concentrations, ethanol can directly erode the gastrointestinal tract, causing acute gastritis and gastric ulcers, which can further result in lesions such as perforation and cancer [3,4,5].

Proton pump inhibitors (PPI) are currently one of the main drugs used to treat gastroesophageal disorders. Among them, omeprazole, lansoprazole, and others are widely used clinical PPI [6]. In addition to PPI, common medications for treating gastric functional disorders include prokinetic agents and H2-receptor antagonists (H2RA) [7]. However, ample evidence now suggests that the medications employed for managing gastroesophageal disorders might come with certain adverse reactions. For instance, the long-term use of PPI and H2RA can affect the body’s absorption of vitamin B12 and increase the risk of developing bacterial peritonitis [8,9]. In summary, it is necessary to seek natural, non-toxic substances that can effectively enhance the gastric mucosal barrier and improve gastrointestinal function, such as polysaccharides and food-derived bioactive peptides; research has shown that wheat peptides and calcitonin-gene-related peptides have protective mechanisms against ethanol-induced gastric ulcers in rats [10,11,12,13].

Walnuts are an important nut product, originally hailing from Southeastern Europe, East Asia, and North America [14]. Walnuts currently rank second in global nut production after almonds, whereas walnut kernels are often regarded as the main oilseed crop during processing, while the by-product of walnut meal is ignored [15]. Walnut meal consists of 40% protein, which contains a notably high content of essential amino acids, making it an excellent source of dietary protein intake [16]. In comparison to walnut protein, its hydrolyzed product, walnut peptides, exhibit superior biological functional properties, such as anti-inflammatory [17], antioxidant [18], antibacterial [19], and neuroprotective effects [20]; furthermore, walnut peptides were confirmed to have a regulatory effect on inflammatory bowel disease (IBD) [21]. However, it is currently unclear whether walnut peptides have a positive impact on damaged gastric mucosal barriers. 

In this research, we examined the influence of walnut peptides (WP) derived from walnut meal on ethanol-induced gastric mucosal injury in mice, elucidating its mechanism of action. An ethanol-induced gastric mucosal barrier model in mice was established, and gastric mucosal damage was observed and pathologically analyzed. The gastric mucosal injury index, injury inhibition rate, antioxidant indices such as aspartate aminotransferase (AST), alanine aminotransferase (ALT), malondialdehyde (MDA), reduced glutathione (GSH), total antioxidant capacity (T-AOC), glutathione disulfide (GSSG) levels, and inflammatory indices including mouse epidermal growth factor (EGF), myeloperoxidase (MPO), mouse tumor necrosis factor-alpha (TNF-α), and mouse interleukin-1β (IL-1β) were determined for evaluating the protective effect of WP against ethanol-induced gastric mucosal injury. Finally, WP was revealed to mediate its biological effects through antioxidant mechanisms by Western blot, offering a theoretical foundation for utilizing walnut meal resources.

## 2. Materials and Methods

### 2.1. Materials and Reagents

Walnut peptides (purity of 99%) were purchased from Shaanxi Xinpai Biotechnology Co., Ltd., Xian, China. Omeprazole was obtained from Renhe (Group) Development Co., Ltd., Beijing, China. MDA, T-AOC, GSH, AST, ALT and GSSG detection kits were purchased from Beijing Solaibao Technology Co., Ltd., Beijing, China. EGF, TNF-α, MPO, and IL-1β ELISA kits were purchased from Wuhan Hualianke Biological Technology Co., Ltd., Wuhan, China. BCA Protein Concentration Determination kits were purchased from Shanghai Biyuntian Biotechnology Co., Ltd., Shanghai, China.

### 2.2. Animals and Groups

Specific pathogen-free (SPF) 4-week-old male Kunming mice were acquired from Beijing Weitonglihua Experimental Animal Technology Co., Ltd., (Beijing, China). After 7 days of acclimatization, they were randomly divided into five groups, blank control group (CK), model group (MG), positive omeprazole control group (PG, 20 mg/kg bw), low-dose walnut peptide group (LWG, 200 mg/kg bw), and high-dose walnut peptide group (HWG, 400 mg/kg bw), with 12 in each group [13]. All animal procedures were in accordance with the Animal Ethics Committee of the Beijing Key Laboratory of Functional Food from Plant Resources (Permit number: A330-2023-2) and conformed to the guidelines for the care and use of laboratory animals set by the National Institutes of Health. LWG and HWG were given samples at a dose of 10 mL/kg bw daily, while CK and MG were gavaged with an equal dose of distilled water. Mouse body weight was measured every three days, with a fixed weighing time, and the final weight measurement was taken before the last gavage. After the final gavage, the mice were fasted for 24 h to ensure complete digestion and absorption of gastric contents, with free access to water during this period. Except for the CK group, all other groups were given 75% ethanol (0.1 mL/20 g) by gavage to induce acute gastric mucosal injury, while the CK group received an equivalent amount of distilled water [22]. One hour after the gavage, the mice were euthanized by dislocating the cervical spine, and organs (liver, kidney, spleen, gastric) and serum were rapidly collected after enucleating the eyeballs and drawing blood from the neck dislocation site.

### 2.3. Peptide Components Analysis of Walnut Peptides Based on LC-MS/MS

An appropriate amount of sample was dissolved in 50 mM NH_4_HCO_3_, and DTT solution was added to the dissolved sample to a final concentration of 10 mmol/L, which was reduced in a 56 °C water bath for 1 h, and alkylated by 50 mM IAM at room temperature in dark for 40 min. The peptide underwent desalting through a self-priming desalting column, and the solvent was removed in a vacuum centrifuge at 45 °C. After reducing alkylation, we used LC-MS/MS (liquid chromatography–tandem mass spectrometry, Thermo Fisher Scientific, Waltham, MA, USA) to analyze the peptide separation and identification, equipped with an Easy-nLC 1200 system (Thermo Fisher Scientific, Waltham, MA, USA) and an Acclaim PepMap RPLC C18 column (size 150 µm × 15 cm, particle size 1.9 µm, Dr. Maisch GmbH, Ammerbuch, Germany). The injection volume was 5.0 µL. The flow rate was 600 nL/min. Solvent A consisted of ultrapure water with 0.1% (*v*/*v*) formic acid, while Solvent B comprised acetonitrile with 0.1% (*v*/*v*) formic acid. The solvent gradient used for the isolation of walnut peptides was as follows: from 4% B to 8% B in 2 min, from 8% B to 28% B in 43 min, from 28% B to 40% B in 10 min, from 40% B to 95% B in 1 min, and finally maintained at 95% B in 10 min. The walnut peptides were sequenced by Beijing Bio-Tech Pack Technology Company Ltd., (Beijing, China).

### 2.4. Organ Index Calculation

After euthanasia of the mice, the viscera (liver, kidney, spleen, and gastric) were removed, the fat was removed, and the organs were washed with saline and drained with filter paper to record the weights. The mice’s organ index was computed using Equation (1):

The organ index (g/g) = organ mass (g)/mouse body weight (g)(1)

### 2.5. Evaluation of Gastric Mucosal Injury

To assess the severity of gastric mucosal lesions, the excised stomachs were dissected along the greater curvature and thoroughly washed with pre-chilled PBS. Subsequently, the emptied and flattened stomach samples were photographed. Following that, the lesion length and width in the flattened stomach samples were measured using a vernier caliper. Briefly, each lesion was scored from 0 to 5 as follows: (a) undetectable lesions (score of zero); (b) bleeding point/pc (score of 1); (c) lesions < 1 mm length (score of 2); (d) lesions of 1–2 mm length (score of 3); (e) lesions of 2–3 mm length (score of 4); (f) lesions > 3 mm length (score of 5), when the width of the lesion exceeded 2 mm, the score was doubled [23]. After washing the gastric specimen, the degree of gastric injury was assessed according to Formulas (2) and (3) as follows:

Gastric Mucosal Injury Index = Bleeding Point Score + Ulcerated Stripe Score(2)

Gastric Mucosal Injury Inhibition Rate = (Model Group Injury Index − Test Substance Group Injury Index)/Model Group Injury Index × 100%(3)

The most severely affected area of the gastric mucosa from each mouse was dissected to obtain tissue samples measuring approximately 5 mm × 5 mm. These tissue samples were then processed through fixation (4% formalin solution), routine gradient dehydration, embedding, sectioning, slide preparation, and hematoxylin and eosin (HE) staining, The HE staining portion was undertaken by Wuhan Seville Biotechnology Co., Ltd. (Wuhan, China). Subsequently, observations were made under an optical microscope. Microscopic damage was scored from 0 to 14, as follows: (a) loss of mucosal epithelium (0–3 points); (b) upper mucosal edema (0–4 points); (c) hemorrhagic damage (0–4 points); and (d) inflammatory cell influx (0–3 points) [24]. The total score, as per the two evaluation criteria mentioned above, was calculated to express the final gastric mucosal tissue injury score.

### 2.6. Determination of AST and ALT Levels in Serum

We adhered to the procedural steps outlined in the commercial assay kits for assessing and analyzing the AST and ALT levels in the serum. The enzyme activities for AST and ALT were quantified in U/mL.

### 2.7. Measurement of Gastric Tissue Antioxidant Capacity

The levels of MDA, T-AOC, GSH, and GSSG were used as indicators of gastric tissue antioxidant capacity. Commercial assay kits (Beijing Soleibao Technology Co., Ltd., Beijing, China) were used to measure these parameters following the manufacturer’s instructions. Gastric tissues were minced with scissors and mixed with pre-cooled phosphate buffer solution (PBS, pH = 7.4) at a 1:9 ratio. The mixture was homogenized using a homogenizer to obtain a 10% gastric tissue homogenate (*w*/*v*). The homogenate was then centrifuged at 5000 rpm at 4 °C for 15 min. The supernatant was collected, and the levels of MDA, T-AOC, GSH, MPO, and GSSG were determined. MDA levels were expressed in nmol/g, T-AOC levels in μmol/g, GSH levels in μg/mg prot, and GSSG levels in μg/mg prot.

### 2.8. Measurement of Gastric Tissue Regulatory Mediator Levels

Enzyme-linked immunosorbent assay (ELISA) kits (Wuhan Hualianke Biological Technology Co., Ltd., Wuhan, China) were used to detect the levels of EGF, MPO, TNF-α, and IL-1β in the supernatant of gastric tissue homogenates, following the manufacturer’s instructions. EGF, TNF-α, and IL-1β levels were expressed in ng/L, MPO levels in ng/mL.

### 2.9. Western Blot Assay

Total protein was extracted from gastric tissues by grinding an appropriate amount of tissue in liquid nitrogen. The gastric tissue homogenate was homogenized in a mixture of RIPA lysis buffer (Shanghai Biyuntian Biological Technology Co., Ltd., Shanghai, China) and protease phosphatase inhibitor cocktail (Beijing Solaibao Technology Co., Ltd., Beijing, China). Protein samples underwent separation using 10% SDS-PAGE, were transferred to a polyvinylidene fluoride (PVDF) membrane, and then blocked with 5% skim milk in Tris-buffered saline with Tween 20 (TBST). Subsequently, the membrane was incubated overnight at 4 °C with anti-HO-1, anti-Keap-1, anti-NQO-1, and anti-Nrf2 antibodies. Following three washes with TBST, the membrane was exposed to HRP-conjugated secondary antibodies at room temperature for a duration of 2 h. Images were acquired using the Image Quant LAS 4000 Mini system (GE Healthcare, Chicago, IL, USA), and grayscale values were measured using Image J 2.0 software.

### 2.10. Statistical Analysis

Statistical analysis was performed using SPSS software (IBM SPSS Statistics 26). Data are presented as the mean ± S.E.M. A one-way analysis of variance (ANOVA) was conducted, followed by Duncan’s test for multiple comparisons, with the significance level set at *p* < 0.05. Graphs were created using GraphPad Prism 8 software, and tables were created using Excel 2021 software. Graphical abstracts drawn by Figdraw.

## 3. Results

### 3.1. Analysis of Walnut Peptide Amino Acid Sequences

LC-MS/MS was used to identify and analyze the amino acid sequences of the main peptide segments in the walnut peptide samples to determine the composition and identification of the major peptide segments in the samples. Table 1 lists the top eight peptide segments with the highest scores, including EIDIGVPDEVGRL, DLAPTHPIRL, LDRLIPVLE, SVIQH, NTGSPITVPVGR, SKRPTF, DREIDIGVPDEVGRL, and RDENEKL. Among them, the highest score was 490.0 for EIDIGVPDEVGRL, indicating that the results of peptide segment identification through LC-MS and secondary spectrum quality integration were the most reliable among all peptide segments. In terms of peptide segment abundance, SKRPTF had the highest abundance at 81,398,000, composed of six amino acids, with an *m*/*z* of 368.211 and a score of 428.9. It had the highest output detected among these eight peptide segments, indicating its high content in the sample. These amino acid sequences may provide potential clues for further research into the mechanisms underlying the impact of walnut peptides on gastric mucosal barrier damage in mice.

### 3.2. The Effects of WP on Mouse Body Weight and Organ Indices

**Figure 1:**
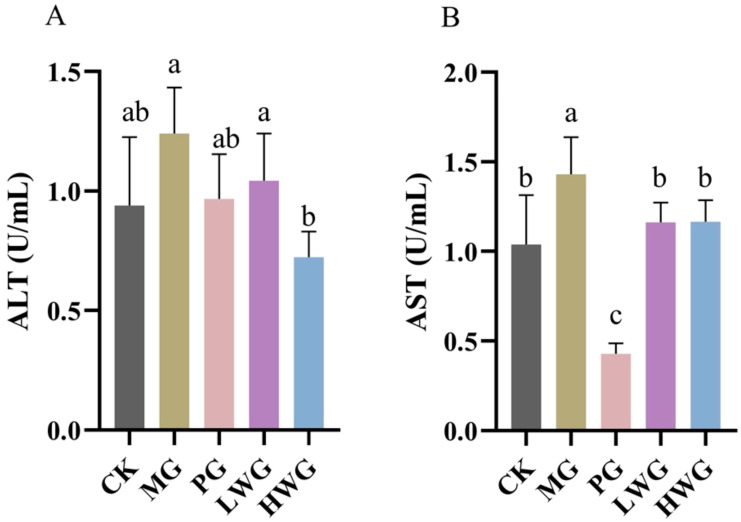
The effects of WP on the serum levels of ALT (**A**) and AST (**B**) in mice. CK group: blank control group; MG group: model group; PG group: positive omeprazole control group (20 mg/kg bw); LWG group: low-dose walnut peptide group (200 mg/kg bw); HWG group: high-dose walnut peptide group (400 mg/kg bw). Different letters represent significant differences between groups at *p* < 0.05.

During the gavage period, mice in all groups showed no abnormal behavior, and there were no significant changes in food and water intake, or signs of hair loss or death. The quality of the mice directly reflected their growth status, as shown in Figure 1. As a result of fasting, all groups experienced a decline in body weights on Day 24 and the trend in the body weight changes in the LWG and HWG groups were similar to the CK group, suggesting that walnut peptides did not have adverse effects on the body weights or the growth status of the mice. As shown in Table 2, the liver indices of the LWG and HWG groups were 42.15 mg/g and 42.08 mg/g, respectively, which were not significant different compared to the PG group (*p* > 0.05), but higher and significantly different from the CK group (*p* < 0.05). The gastric indexes of the LWG, HWG, and PG groups were 8.92 mg/g, 8.99 mg/g, and 8.67 mg/g, respectively, which were not significantly different compared to the CK and MG groups (*p* > 0.05). However, the gastric index was the lowest in the CK group and highest in the MG group. Similarly, the kidney indexes and spleen indexes of the LWG and HWG groups were not significantly different (*p* > 0.05) compared to the CK group. This indicates that the effect of the walnut peptides on the mice organ coefficients was similar to that of the positive drug and did not cause damage to the internal organs of the mice and did not form a significant effect on the organ coefficients of the mice.

### 3.3. The Effects of WP on Serum ALT and AST Levels

The ALT levels were 0.72 U/mL for HWG, 0.94 U/mL for CK, and 0.967 U/mL for PG. According to Figure 1A, it is evident that the ALT levels in HWG were significantly reduced compared to MG (*p* < 0.05) and showed no significant difference compared to CK and PG (*p* > 0.05). The AST levels of LWG and HWG were 1.16 U/mL and 1.17 U/mL, as shown in Figure 1B, compared to the MG group, the AST levels in the WP treatment groups all exhibited a significant decrease from the MG group (*p* < 0.05) and showed no significant difference compared to the CK group (*p* > 0.05).

### 3.4. The Impact of WP on Gastric Mucosal Tissue Pathological Changes

**Figure 2:**
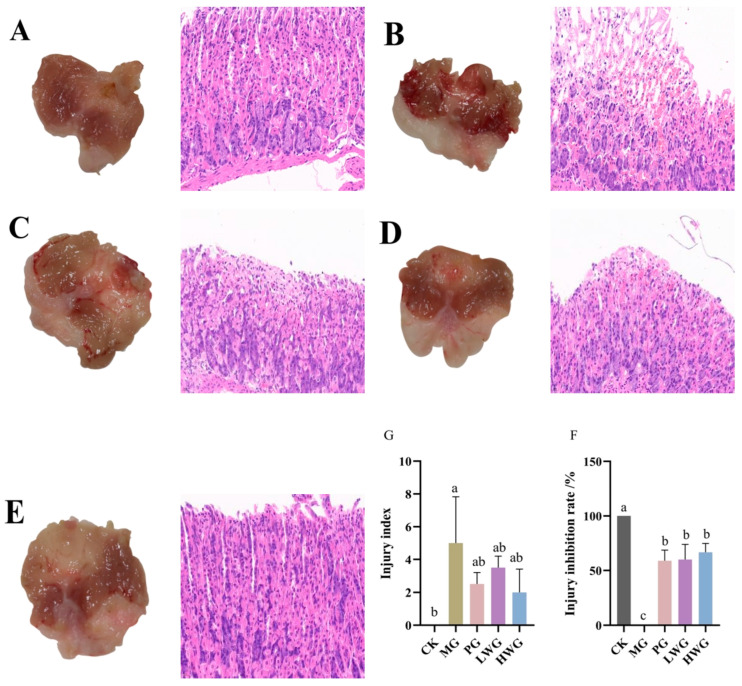
The effects of WP on gastric mucosal injury in mice. From left to right: Observation of gastric mucosal pathological features through gross morphology and HE staining (scale bar: 20 μm). (**A**) CK group. (**B**) MG group. (**C**) PG group. (**D**) LWG group. (**E**) HWG group. (**F**) Injury inhibition rate in each group of mice. (**G**) Injury index in each group of mice. CK group: blank control group; MG group: model group; PG group: positive omeprazole control group (20 mg/kg bw); LWG group: low-dose walnut peptide group (200 mg/kg bw); HWG group: high-dose walnut peptide group (400 mg/kg bw). Different letters represent significant differences between groups at *p* < 0.05.

According to Figure 2, in the CK group, the gastric mucosa of mice appeared intact with normal color and shape, and no obvious signs of hemorrhage, ulceration, or erosion were observed, whereas the gastric mucosa of mice in the MG group showed obvious hemorrhagic damage, with the appearance of multiple large hemorrhagic streaks and hemorrhagic dots, which confirms that the model of ethanol-induced gastric mucosal injury was successfully established. The gastric mucosa of the PG group showed a small number of hemorrhagic spots but no ulceration or erosion, and the degree of injury was significantly reduced compared with that of the MG group, with an inhibition rate of 61.33%; in the LWG and HWG groups, the gastric mucosa of mice also showed a few bleeding spots, but no erosion or ulceration, with an inhibition rate of 58.67% and 66.67%. Compared with the PG group, there was no significant difference in the damage inhibition rate (*p* > 0.05); however, there was a significant difference compared to the MG group (*p* < 0.05). The HE staining results revealed that in the CK group, the gastric mucosal epithelium had a well-organized glandular structure, and the mucosal layer, submucosal layer, and muscular layer appeared intact, well-structured, and free from obvious edema and inflammation infiltration. In the MG group, the glandular structure of the gastric tissue was disrupted, with localized epithelial cell necrosis, shedding, and partial mucosal bleeding, confirming the successful modeling of the gastric mucosal injury induced by ethanol. In the PG, LWG, and HWG groups, there were a noticeable improvement in the status of the gastric mucosal injury. The gastric mucosal injury indices in the PG, LWG, and HWG groups were 2.5, 3.5, and 2, respectively, with no significant difference among these three groups (*p* > 0.05), and significantly different from the MG group (*p* < 0.05).

### 3.5. The Effects of WP on Gastric Tissue Oxidative Stress

**Figure 3:**
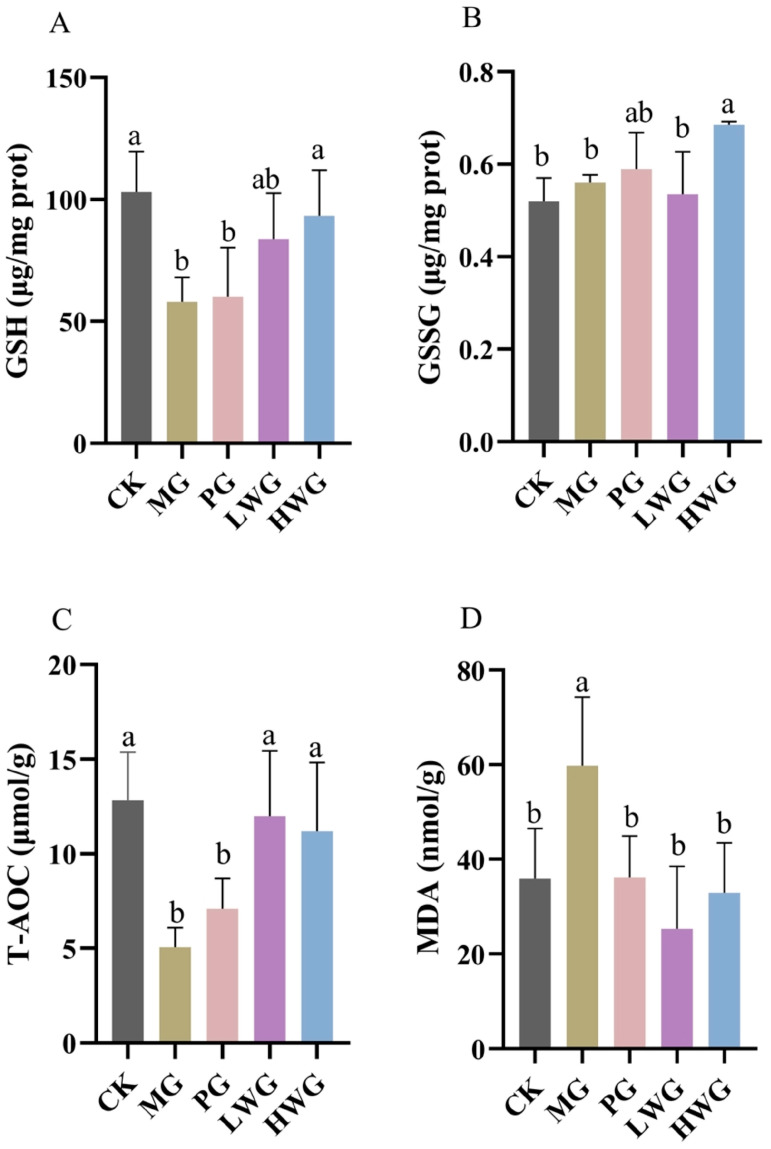
The effects of WP on gastric tissue oxidative stress markers of GSH (**A**), GSSG (**B**), T-AOC (**C**), and MDA (**D**) in mice. CK group: blank control group; MG group: model group; PG group: positive omeprazole control group (20 mg/kg bw); LWG group: low-dose walnut peptide group (200 mg/kg bw); HWG group: high-dose walnut peptide group (400 mg/kg bw). Different letters represent significant differences between groups at *p* < 0.05.

We investigated the effects of WP on relevant oxidative stress markers in ethanol-induced gastric injury in mice using an assay kit. According to Figure 3, we observed that the presence of WP effectively decreased the content of MDA and significantly increased the content of GSH, T-AOC, and GSSG. The GSH contents of the LWG and HWG groups were 83.86 μg/mg prot and 93.26 μg/mg prot, which were not significantly different from the CK group (*p* > 0.05), but formed a significant difference with the MG group (*p* < 0.05). The GSSG content of the HWG group was 0.69 μg/mg prot, which formed a significant difference with the MG group (*p* < 0.05). The T-AOC contents of the LWG, HWG, and CK groups were 11.99 μmol/g, 11.20 μmol/g, and 12.83 μmol/g, and these three results were not significantly different but formed a significant difference with the MG group (*p* < 0.05). The MDA content of 59.81 nmol/g in the MG group formed a significant difference with the LWG, HWG, and CK groups, which were 25.32 nmol/g, 32.93 nmol/g, and 35.93 nmol/g (*p* < 0.05).

### 3.6. The Effects of WP on Gastric Tissue Inflammation

**Figure 4:**
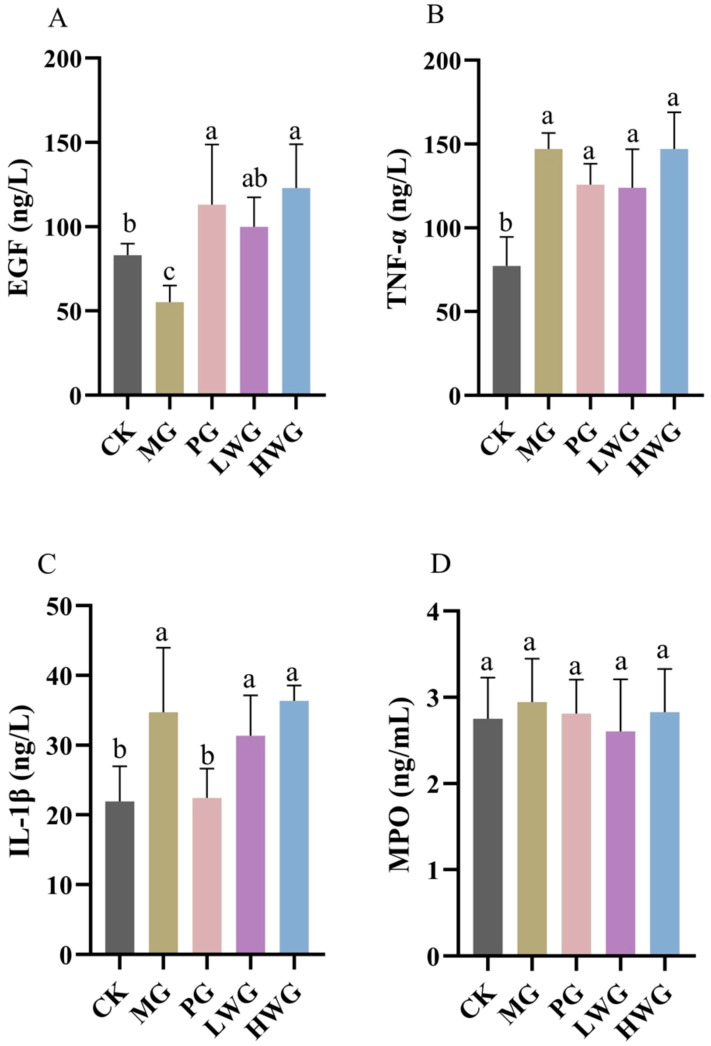
The test results of WP on the expression levels of inflammatory factors in gastric tissue of EGF (**A**), TNF-α (**B**), IL-1β (**C**), and MPO. (**D**) in mice. CK group: blank control group; MG group: model group; PG group: positive omeprazole control group (20 mg/kg bw); LWG group: low-dose walnut peptide group (200 mg/kg bw); HWG group: high-dose walnut peptide group (400 mg/kg bw). Different letters represent significant differences between groups at *p* < 0.05.

We investigated the impact of WP on the gene expression of pertinent inflammatory factors in ethanol-induced gastric injury in mice through ELISA. According to Figure 4, we observed that WP significantly increased the expression level of EGF, which was 99.99 ng/L in the LWG group, 122.89 ng/L in the HWG group, and 103.80 ng/L in the PG group, with no significant difference among the three groups (*p* > 0.05), and was significantly different from the MG group (*p* < 0.05). Although the expression levels of TNF-α and IL-1β in the LWG group were slightly lower than those in the MG group, the differences were not statistically significant (*p* > 0.05). The results of the MPO assay showed no significant differences between the groups (*p* > 0.05). 

### 3.7. The Effects of WP on the Protein Expression of Inflammatory Factors in Gastric Tissue

The Western blotting (WB) results showed that the Nrf2 expression of the HWG group was 0.82; the difference was not statistically significant compared to the PG group, which was 0.99 (*p* > 0.05), but significantly different from MG group, which was 0.41 (*p* < 0.05), proving that a high dose of WP could effectively up-regulate the expression of Nrf2. The expression levels of Keap1 in all groups were not statistically significant (*p* > 0.05).

To further confirm that Nrf2 is a key target for antioxidant stress and the inhibition of iron metamorphosis, we explored the concentrations of two crucial downstream target genes of Nrf2, namely HO-1 and NQO1. The HO-1 gene expression of the LWG group was 1.27, which was significantly different from that of MG group, which was 0.51 (*p* < 0.05). NQO1 gene expression of the LWG and HWG groups were 1.08 and 1.03, which were significantly different from the MG group content of 0.77 (*p* < 0.05) and not significantly different from the PG group content of 1.23 (*p* > 0.05). In summary: the WB results indicated that the expression of two key downstream target genes, HO-1 and NQO1, was significantly increased in the presence of WP.

## 4. Discussion

The eight highest scoring peptides, EIDIGVPDEVGRL, DLAPTHPIRL, LDRLIPVLE, SVIQH, NTGSPITVPVGR, SKRPTF, DREIDIGVPDEVGRL, and RDENEKL, were obtained from the LC-MS/MS results. Among them, the highest scoring peptide EIDIGVPDEVGRL has a C-terminal of Leu and an N-terminal of Glu, and this peptide was detected to be derived from the A0A2I4EK72 protein of walnut by searching through the Uniprot Whole Species Sequence Library (https://www.uniprot.org, accessed on 12 September 2023). The most abundant of the eight proteins was a hexapeptide, SKRPTF, with a C-terminal of Phe and an N-terminal of Ser, which was searched to be derived from a walnut-derived RING-type E3 ubiquitin transferase. The determination of the peptide composition and the analysis of the amino acid sequence of the sample can help to provide theoretical and experimental data support for the subsequent active peptides of walnut peptides that contribute significantly to the protection of gastric mucosa. Ethanol exhibits corrosive effects on the gastric mucosa, disrupting the surface mucus layer and mucous cells, thereby weakening the protective role of gastric mucosal defense factors [25]. Research has shown that gavage with 75% ethanol on empty gastric tissue can lead to gastric mucosal vascular occlusion, impaired blood circulation, cell necrosis, and bleeding in mice [26]. These results align with the findings of our study. Furthermore, our research indicates that different doses of walnut peptides can significantly reduce ethanol-induced gastric mucosal damage in mice, as evidenced by a significant decrease in gross observations and pathological tissue damage scores, along with an increase in the injury inhibition rate.

Ethanol-induced liver injury manifests with the release of hepatic enzymes (ALT and AST) into the circulatory system. Elevated serum levels of ALT and AST signify damage to cell membranes and cellular mitochondria in the liver, respectively [27]. The experimental findings revealed that ethanol induced harm to the cell membrane of the liver, but the presence of WP inhibited the excessive release of ALT and AST, thus reducing the damage of alcohol to the liver cells and restoring the AST indexes to almost normal levels, and a high concentration of WP could restore the ALT indexes to almost normal levels, which is consistent with the results of a previous similar study on dendrobium officinale [28].

Gastric tissues undergo a series of oxidative stress responses when stimulated by ethanol. In this experiment, the expression levels of GSH, GSSG, T-AOC, and MDA were used to assess the antioxidant capacity of mice. GSH is the major source of thiol groups in activated gastric mucosal cells and plays a role in maintaining the normal redox state of intracellular protein thiols. It is also an important antioxidant in cellular physiological activities [29]. Glutathione is converted to GSSG by glutathione peroxidase, which is involved in scavenging free radicals. When gastric mucosal cells accelerate the accumulation of hydrogen peroxide and lipid peroxides, the activity of glutathione peroxidase decreases, leading to a decrease in the level of GSSG expression, which can exacerbate the damage [30]. The results showed that ethanol-induced impairment of the gastric mucosal barrier decreased the expression of GSH and GSSG, while the presence of WP promoted the expression of GSH and GSSG, which reduced the excessive damage caused by oxidative stress. T-AOC is one of the main measurements used for assessing and evaluating the level and potential of oxidative stress in certain diseases [31]. The experimental results showed that when ethanol caused gastric mucosal barrier damage, the T-AOC values of mice decreased significantly, and the presence of WP could promote the expression of T-AOC and restore it to normal levels. MDA is an important indicator for assessing lipid peroxidation when reactive oxygen species interact with unsaturated fatty acids [32]. The experimental results showed that the MDA content in mice increased significantly when ethanol caused gastric mucosal barrier damage, and the presence of WP prevented the excessive accumulation of MDA. It has been demonstrated that wheat peptides can downregulate MDA levels in mice with alcoholic gastric ulcers, and our findings are consistent with them [5,33]. In summary, WP can alleviate the oxidative stress of alcohol-induced gastric mucosal injury by promoting the expression of GSH, GSSG, and T-AOC and reducing the expression of MDA. 

When ethanol enters the digestive tract and stimulates gastric mucosal damage, it involves the interaction of various inflammatory cytokines. During the inflammatory process, a large amount of reactive oxygen species is generated at the inflammatory site, exacerbating oxidative stress reactions and causing tissue damage [34]. In this experiment, the expression levels of EGF, IL-1β, TNF-α, and MPO were used to evaluate the anti-inflammatory effects of walnut peptides. EGF is a growth factor that can inhibit gastric acid secretion, promote the proliferation of gastric mucosal epithelial cells, and facilitate tissue healing. When gastric tissue is damaged, the secretion of EGF is inhibited [35]. MPO is a peroxidase produced by neutrophils, which can reflect the number of neutrophils and the degree of inflammation infiltration in the gastric mucosa. It can also indicate the extent of damage caused by inflammation [36]. TNF-α can promote the production of various inflammatory factors and inhibit the blood circulation around the damaged mucosa, thereby delaying healing. Under normal conditions, the expression of TNF-α in tissues is low. When there is damage, the tissue secretes a large amount of TNF-α, worsening the injury [37]. IL-1β is a pro-inflammatory cytokine released by immune cells, and its expression level is strongly associated with intense gastric inflammation, the progression of gastric cancer, and an unfavorable prognosis. This may be because IL-1β has a strong inhibitory effect on parietal cells that induce gastric acid secretion and can drive the overexpression of several pro-inflammatory mediators, thereby altering the gastric mucosa’s homeostasis [38,39]. However, this experimental study demonstrated that there was no significant difference in the expression levels of TNF-α, IL-1β, and MPO between the WP experimental group and the MG group. This indicated that the presence of WP did not alleviate the increased expression levels of the inflammatory factors TNF-α, IL-1β, and MPO caused by damage to the gastric mucosal barrier, which proved that WP relied on the increase in the secretion level of EGF to alleviate the effect of ethanol on the gastric mucous membrane damage; this aligns with the findings of a prior analogous study on wheat peptides and fucoidan [40].

**Figure 5:**
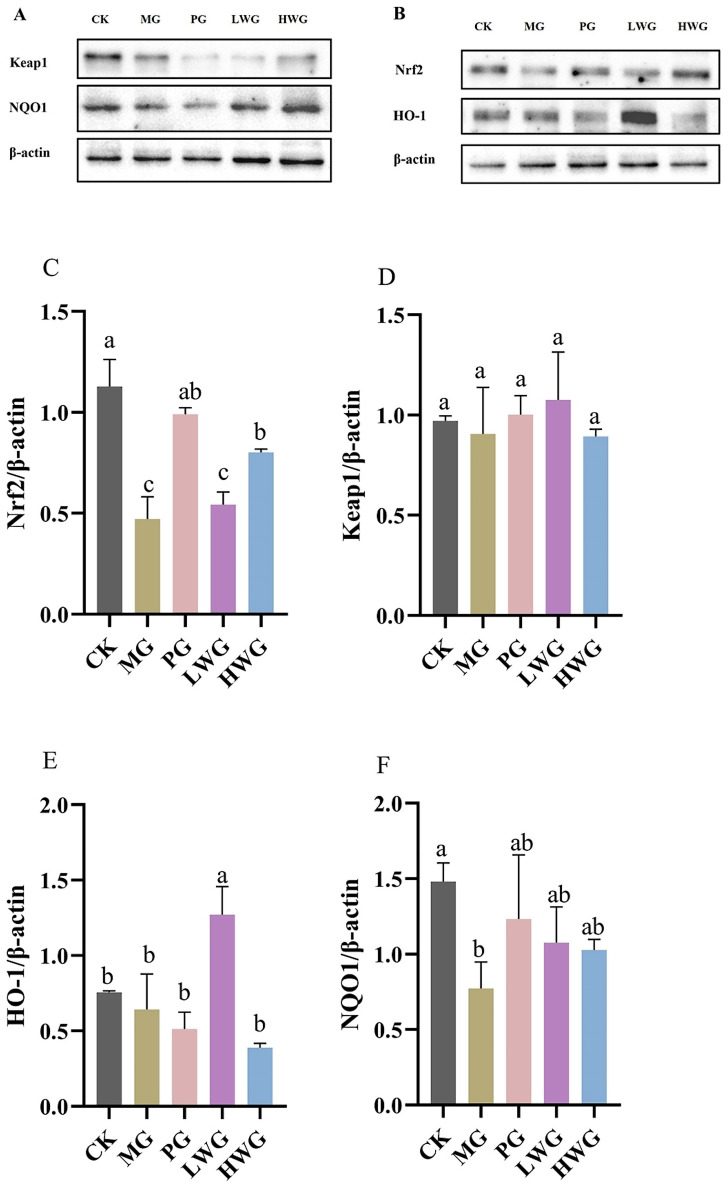
The effects of WP on the protein expression of inflammatory factors in gastric tissue. Protein blot analysis of Nrf2, NQO, Keap1, and HO-1. (**A**,**B**) Protein content analysis of Nrf2, Keap1, HO-1, and NQO1 (**C**–**F**). CK group: blank control group; MG group: model group; PG group: positive omeprazole control group (20 mg/kg bw); LWG group: low-dose walnut peptide group (200 mg/kg bw); HWG group: high-dose walnut peptide group (400 mg/kg bw). Different letters represent significant differences between groups at *p* < 0.05.

**Figure 6:**
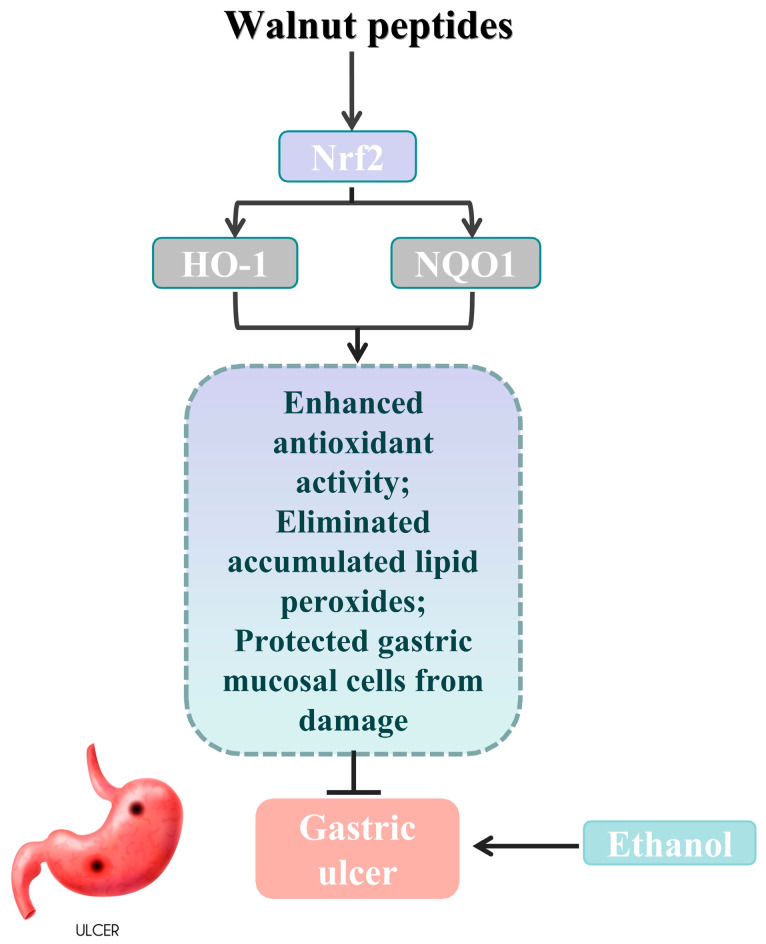
Schematic abstract: WP enhanced the gastric mucosal barrier by activating Nrf2 signaling, as well as increasing the expression of HO-1 and NQO1, thereby ameliorating oxidative stress and ethanol-induced gastric mucosal damage in mice (gastric ulcer image sourced from freepik).

An increasing body of experiments demonstrates the significant role of the Nrf2/Keap1 signaling pathway in protecting the gastric mucosa from oxidative stress [41]. HO-1 and NQO1 are key downstream target genes of Nrf2, and studying the gene expression levels of HO-1 and NQO1 can help to validate Nrf2 as a critical target in antioxidant stress [42]. To further investigate the protective effect of WP against ethanol-induced oxidative stress in mouse gastric mucosal histiocytes, the activity of Nrf2, Keap1, HO-1, and NQO1 enzymes related to oxidative stress in the cytoplasm of gastric histiocytes was determined (Figure 5). Experiments have shown that ethanol-induced gastric mucosal damage in mice leads to the suppression of HO-1, NQO1, and Nrf2 expression. However, HO-1 and NQO1 were significantly upregulated in the LWG group compared to the MG group. However, HO-1 and NQO1 were significantly upregulated in the LWG group compared to the MG group. Similarly, Nrf2 in the HWG group had a trend of significant upregulation, with the same trend as in the CK and PG groups. And there was no notable variance in Keap1 expression between the groups. It was demonstrated that the presence of WP could alleviate this inhibition, leading to an enhancement in antioxidant activity and the removal of accumulated lipid peroxides, consequently shielding gastric mucosal cells from harm (Figure 6).

## 5. Conclusions

In summary, our study demonstrates the potential therapeutic value of walnut peptides in protecting against ethanol-induced gastric mucosal barrier damage in mice. We have investigated the antioxidant and anti-inflammatory mechanisms of walnut peptides during this process. This opens up new avenues for exploring non-toxic and side-effect-free natural products as alternatives to existing drugs that may improve gastric mucosal barrier damage but carry potential risks.

## Figures and Tables

**Table 1:** The amino acid sequence of walnut peptides.

Peptide Sequence	Observed *m*/*z*	Score	Scan Time	Protein Name	Intensity
EIDIGVPDEVGRL	706.372	490.0	48.0578	A0A2I4EK72	60,222,000
DLAPTHPIRL	566.827	474.2	29.0392	A0A2I4EB92	22,166,000
LDRLIPVLE	534.326	466.0	45.2099	A0A2I4EGJ3	45,179,000
SVIQH	625.331	431.2	7.1603	A0A2I4FTY2	40,419,000
NTGSPITVPVGR	599.334	428.9	25.3499	A0A2I4GH77	25,614,000
SKRPTF	368.211	428.9	8.6987	A0A2I4EFN4	81,398,000
DREIDIGVPDEVGRL	841.940	422.2	43.2557	A0A2I4EK72	13,398,000
RDENEKL	452.230	420.2	7.1808	A0A2I4E5L6	71,960,000

**Table 2:** Organ indexes of mice in each group. CK group: blank control group; MG group: model group; PG group: positive omeprazole control group (20 mg/kg bw); LWG group: low-dose walnut peptide group (200 mg/kg bw); HWG group: high-dose walnut peptide group (400 mg/kg bw). Different letters represent significant differences between groups at *p* < 0.05.

Group	Liver Index (mg/g)	Kidney Index (mg/g)	Spleen Index (mg/g)	Gastric Index (mg/g)	Starting Weight (g)	Final Weight (g)
CK	39.43 ± 2.32 b	12.17 ± 1.41 ab	29.29 ± 1.50 a	7.85 ± 1.82 a	39.05 ± 0.86 a	43.80 ± 2.04 a
MG	42.81 ± 1.96 a	11.95 ± 1.19 ab	35.28 ± 2.16 a	9.36 ± 1.73 a	38.32 ± 1.15 a	42.25 ± 1.97 a
PG	43.48 ± 3.65 a	13.41 ± 2.97 a	28.25 ± 2.26 a	8.67 ± 2.43 a	40.07 ± 1.61 a	44.43 ± 2.22 a
LWG	42.15 ± 3.10 a	11.61 ± 1.31 b	25.38 ± 0.79 a	8.92 ± 1.24 a	38.93 ± 2.41 a	43.97 ± 2.51 a
HWG	42.08 ± 2.95 a	11.87 ± 1.10 ab	24.40 ± 0.62 a	8.99 ± 1.91 a	39.47 ± 1.69 a	44.53 ± 1.11 a

## Data Availability

Data are contained within the article.
